# Integrating Telehealth for Strengthening Health Systems in the Context of the COVID-19 Pandemic: A Perspective from Peru

**DOI:** 10.3390/ijerph20115980

**Published:** 2023-05-28

**Authors:** Walter H. Curioso, Lelis G. Coronel-Chucos, Milagro Henríquez-Suarez

**Affiliations:** 1 Vicerrectorado de Investigación, Universidad Continental, Lima 15046, Peru; 2 Health Services Administration, Continental University of Florida, Margate, FL 33063, USA; 3 Facultad de Ciencias de la Salud, Universidad Continental, Lima 15046, Peru; 4 Rectorado, Universidad Nacional Autónoma Altoandina de Tarma, Junin 12651, Peru

**Keywords:** telehealth, health-information systems, digital health, telemedicine, open data, healthcare centers, Peru

## Abstract

The COVID-19 pandemic forced the government to rapidly modify its legal framework to adopt telemedicine and promote the implementation of telehealth services to meet the healthcare needs of patients in Peru. In this paper, we aim to review the main changes to the regulatory framework and describe selected initiatives to promote the telehealth framework that emerged in Peru during the COVID-19 pandemic. In addition, we discuss the challenges to integrate telehealth services for strengthening health systems in Peru. The Peruvian telehealth regulatory framework began in 2005, and in subsequent years, laws and regulations were established that sought to progressively implement a national telehealth network. However, mainly local initiatives were deployed. In this sense, significant challenges remain to be addressed, such as infrastructure in healthcare centers, including high-speed Internet connectivity; infostructure of health-information systems, including interoperability with electronic medical records; monitoring and evaluation of the national agenda for the health sector in 2020–2025; expanding the healthcare workforce in terms of digital health; and developing the capacities of healthcare users on health literacy, including digital aspects. In addition, there is enormous potential for telemedicine as a key strategy to deal with the COVID-19 pandemic and to improve access to rural and hard-to-reach areas and populations. There is thus an urgent need to effectively implement an integrated national telehealth system to address sociocultural issues and strengthen the competencies of human resources in telehealth and digital health in Peru.

## 1. Introduction

The COVID-19 pandemic has resulted in a rapid expansion of digital health services globally, including telehealth [1]. Digital health applications and devices (including telemedicine) have been applied to support the management and control of the COVID-19 pandemic, including patient triage, public health, disease surveillance, contact tracing, supply-chain management, patient diagnosis and monitoring, healthcare training and support, and public-health and biomedical research [2].

According to the World Health Organization, telemedicine is defined as “the delivery of health-care services by all health-care professionals using information and communication technologies for the exchange of valid information for diagnosis, treatment and prevention of disease and injuries” [3]. With the advancement of electronic and mobile technologies, as well as the rapid implementation of medical devices, telemedicine is becoming more accessible and useful in many areas of medicine specialties, such as pneumology, cardiology, urology, infectious disease, and rheumatology, among others [1]. Telemedicine has been recognized as a method to protect both infected and non-infected individuals, as well as healthcare personnel. In addition, telemedicine presents a method of triage for COVID-19 patients without the need to physically attend a healthcare center [1].

Given the lockdown established by governments in many countries in the world, telemedicine allowed mildly infected and non-infected physicians to continue practicing from their homes [1]. Telemedicine use during the COVID-19 pandemic was beneficial for patient diagnosis, treatment, and follow-up and was also beneficial for healthcare providers [1].

Despite the fact that telemedicine was already in use prior to COVID-19 in many countries around the world, Latin America’s adoption of telemedicine was extremely low compared to high-income countries [1]. However, an increase in telehealth services in Latin America due to the COVID-19 pandemic was seen despite many barriers, such as technological, educational, infrastructure, legal, and economical barriers, as well as regulations affecting insurance companies regarding telemedicine services, among others [1]. A 2019 study by LeRouge et al. highlighted that financial resources, biases, and physician resistance were the main barriers affecting this slow implementation in Latin America [4].

According to Nittari et al., teleconsultations increased in Latin America, as patients were reluctant to visit in-person healthcare centers due to fears concerning the pandemic [1]. However, many barriers still need to be overcome for the telemedicine system to function properly and effectively in Latin America and around the world [1].

In this paper, we aim to review the main changes to the regulatory framework and describe selected initiatives to promote telehealth that emerged in Peru during the COVID-19 pandemic. In addition, we discuss the challenges to integrating telehealth services for strengthening health systems in Peru.

## 2. The Role of the Regulatory Framework as a Driver for the Adoption of Telehealth in Peru

Several countries in Latin America, such as Peru, modified their legal framework to promote telehealth as a means to provide healthcare access in the context of the mandatory lockdown [5,6,7].

The history of the implementation of telehealth in Peru goes back to 2005, when the first National Telehealth Plan was approved by Supreme Decree 028-2005-MTC [8]. This document was the first multisectoral effort to overcome the gaps in access to health services, especially in remote and rural areas [8]. It is important to point out that this national plan was led by the Ministry of Transport and Communications and not the Ministry of Health. This plan was promoted by the National Telehealth Commission, established in April 2003 by Supreme Decree No. 009-2003-MTC, and a number of public institutions actively participated, including the Ministry of Health, the Ministry of Transport and Communications, the National Institute for Telecommunications Research and Training (INICTEL), Social Security (EsSalud), the Supervisory Agency for Private Investment in Telecommunications (OSIPTEL), and the National Institute of Statistics and Information Technology (INEI). At that time, telehealth was defined as a health service that uses information and communication technologies to become more accessible to consumers and healthcare providers in rural areas or accessible with few interventions.

In 2008, the Telehealth Technical Standard was approved by Ministerial Resolution No. 365-2008 by the Ministry of Health [9]. The resolution involved three axes: (1) provision of health services (telemedicine), (2) management of health services, and (3) information, education, and communication for the population and health personnel.

By 2012, the Conceptual Framework for the Strengthening of Information Systems and Information and Communication Technologies at the Ministry of Health was approved [10]. This document indicates that telehealth constitutes an important element of the health system’s conceptual framework [11]. In addition, the main components to be considered for a telehealth project in the country are established, namely, infrastructure; infostructure; planning, monitoring, and evaluation; governance and leadership; and people and capacity building [11]. Those components were later included in the architectural model for the Peruvian Digital Agenda of the Health Sector 2020–2025, which is well known as the “digital house” [12].

Subsequently, in 2016, Law No. 30421 (the Telehealth Framework Law) was approved [13] and included the following principles and definitions that support telehealth:

(a) Universality: “Through telehealth services, the access to health services for the whole population is guaranteed”.
(b) Equity: “Telehealth services are provided with the same quality and similar options to the population, reducing the existing gap in their access”.
(c) Efficiency: “The resources of the national health system are used rationally, optimizing care in health services through the diverse options of telehealth”.
(d) Quality of service: “Through telehealth services, an improvement in the quality of health is promoted and the capacities of health personnel are strengthened, including user satisfaction”.
(e) Decentralization: “Telehealth is a strategy for the use of health resources that optimizes care in health services, strengthening the decentralization process of the national health system using information and communication technologies”.
(f) Social development: “Through telehealth services, the development of society is promoted, allowing the population to have greater access to health information, knowledge and health rights, promoting the empowerment of people as main subjects of care of their own health, their family and their community, generating new scenarios for citizen participation”.

Although the Telehealth Framework Law (No. 30421) was passed in April 2016 [13], its regulations were approved after almost three years. This law established the general guidelines for the implementation and development of telehealth in Peru, using information and communication technologies to overcome healthcare gaps, with an emphasis on rural areas.

One of the most important milestones at the Peruvian Ministry of Health was the creation of the General Directorate of Telehealth, Referrals, and Emergencies (2017), which included a modification to the organization flowchart of the Ministry of Health. This modification was approved by Supreme Decree No. 008-2017-SA [14]. This Directorate is responsible for formulating and implementing the telehealth policies in the health sector. Its functions are to propose, coordinate, supervise, and evaluate the telehealth sector’s policies. The General Directorate includes the Directorate of Telemedicine, which oversees formulating and implementing standards, plans, strategies, and guidelines for the implementation and management of telemedicine in the health sector, such as the provision of remote services regarding health promotion, prevention, diagnosis, recovery, or rehabilitation, as well as follow-up and monitoring, and includes an educational-training component for health personnel [14].

The Telehealth Framework Law was modified by Legislative Decree No. 1303 [15] (passed in December 2016) and broadened the concept of telehealth, defining it as “a distance health service provided by competent health personnel, through information and communication technologies, to ensure that these services and their related services are accessible mainly to users in rural areas or with limited resolution capacity. The telehealth services are carried out considering the following axes: provision of health services; management of health services; information, education, and communication in health for the population; and capacity building for the health personnel” [15].

In Peru, before the COVID-19 pandemic, the legal framework only regulated consultation activities between a health personnel (who requests the consultation) and a health professional (who responds to the consultation request remotely). The Peruvian regulation established that telehealth services were restricted to healthcare centers, and users could not receive assistance directly from a healthcare provider [6].

In the context of the COVID-19 pandemic, Supreme Decree No. 013-2020-SA was published in April 2020 and stated that the Ministry of Health would establish procedures to provide telemedicine services, with special emphasis on remote medical teleguidance, telemonitoring, and mental health during the health emergency [16].

In May 2020, the Peruvian Ministry of Health approved a legal framework (Legislative Decree No. 1490) that regulated and allowed patients to contact health professionals remotely through telehealth services [17]. This regulation modified the definitions of telehealth and telemedicine [17]. In this sense, telehealth was defined as a remote health service provided by competent health personnel, through information and communication technologies, in a timely and accessible manner for the population [17], whereas telemedicine corresponded to the provision of remote health services in the components of promotion, prevention, diagnosis, treatment, recovery, rehabilitation, and palliative care provided by health personnel who use information and communication technologies [17].

Legislative Decree No. 1490 detailed the types of telemedicine (teleconsultation, teleinterconsultation, teleguidance, telemonitoring, and others) that can be established by the Ministry of Health [17]. Similarly, Legislative Decree No. 1490 stated that electronic prescriptions are to be incorporated into telemedicine services (i.e., authorizing the electronic transmission of medical prescriptions), as well as electronic medical records as a technological tool that allows communication through information and communication technologies [17]. In addition, Legislative Decree No. 1490 also established that the provision of telemedicine services requires the informed consent of the patient [17]. The provision of telehealth services is carried out within the protection of personal-data regulations, as well as considering the issues of security and confidentiality.

Finally, the Decree established the creation of the National Telehealth Network, which involves healthcare facilities and the processes, personnel, and information and communication technologies that provide telehealth services to the population [17]. The management, regulation, articulation, and evaluation of the National Telehealth Network is the responsibility of the Ministry of Health [17]. The website of the National Telehealth Network can be found at following link: https://www.gob.pe/telesalud (accessed on 1 March 2023).

Based on the above regulations and the activities developed by health-related institutions, several telehealth services in the country were implemented [6,7,18]. However, it is necessary to articulate all these regulations with the health system, especially with primary-healthcare centers and the integrated network of health services, to fully immerse telehealth services into the Peruvian healthcare landscape.

## 3. The Role of Telehealth in the Context of the COVID-19 Pandemic in Peru

Countries around the world have developed and scaled up their telemedicine applications due to the lockdowns necessitated by the COVID-19 pandemic [19]. In the health sector, telemedicine is an important aspect for both patients and healthcare professionals to provide access via online consultations and other types of telehealth services, such as telemonitoring, telediagnosis, and tele-education [20].

**Figure 1:**
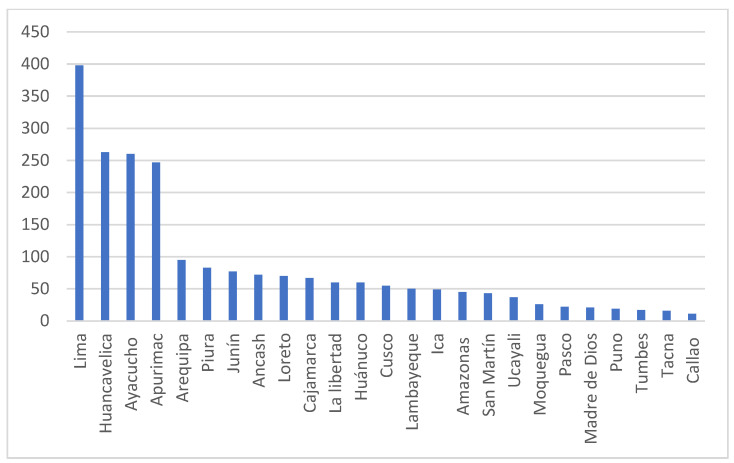
Number of healthcare centers that implemented telehealth services in Peru during the COVID-19 pandemic (data source: Ministry of Health [22]).

The implementation of telehealth services implies not only updating infrastructure with certain technologies [21] but also the infostructure of health-information systems, regulatory frameworks related to telehealth and digital health, the training of health professionals on digital health and health management, and data-science management, as well as addressing leadership and administrative issues for improving telehealth in Peru. [6,11]. The number of healthcare centers that implemented telehealth services in Peru during the beginning of the COVID-19 pandemic [22] is presented in Figure 1. Most telehealth services are provided in Lima, the capital of Peru.

In addition, the National Institute of Statistics and Informatics (INEI) published the technical report Statistics of the Information and Communication Technologies in Households based on the results of the National Households Survey (ENAHO), which is a census-type national survey that is conducted yearly by the INEI. A recent 2022 report (April–June) stated that mobile phones are the main instrument for Internet access in Peru [23]. Among the Peruvian population above 6 years old that has access to the Internet, 67% had access to the Internet only through a mobile phone, 18.6% from a fixed connection at home and a mobile phone, 4.9% only from home, and 3.3% from home, work, and a mobile phone (Table 1).

## 4. Methods

We conducted a narrative review of studies to chart the landscape of telehealth in Peru across the regulatory framework, initiatives that emerged during the COVID-19 pandemic, and the challenges faced in integrating telehealth services in order to strengthen health systems in Peru. As Greenhalgh et al. pointed out, “the narrative review structure selects evidence judiciously and purposively with an eye to what is relevant” [24].

In this sense, we conducted a keyword-search-based literature review to acquire relevant scientific documents (mainly peer-reviewed articles and theses) using Scopus, Web of Science, and SciELO and repositories such as ALICIA and LA Referencia to search for studies with titles or abstracts containing the following keywords: “telehealth”, “telemedicine”, “telediagnosis”, “telemonitoring”, “teleconsultation”, “teleorientation”, “tele-education”, “teletraining”, “telemanagement”, “teletriage”, “remote consultation”, and “Peru”. We did not apply language filters, and we applied a snowballing search methodology using the references cited in the articles identified in the literature search.

The selection of the studies was carried out in two phases. First, all relevant studies were identified from the search terms in the electronic databases by reading titles and abstracts. Subsequently, duplicate studies were eliminated, and the inclusion and exclusion criteria were applied. We considered including studies reporting on the implementation and/or evaluation of telehealth interventions in Peru during the COVID-19 pandemic, published from March 2020 to February 2023, regardless of the language in which they were published. We excluded studies that did not specifically address telehealth as an intervention or primary research focus, studies that were not available in full text, and letters to the editor. Study selection was performed by two independent reviewers (WHC and LCC); any discrepancies were resolved by consensus. The initial search yielded 72 articles and 4 theses. After duplicated articles were removed, 61 articles remained for title and abstract screening. After screening and full-text review, 11 articles and 1 thesis (12 studies) were included in the final review.

## 5. Results

### 5.1. Selected Telehealth Projects and Programs in the Context of the COVID-19 Pandemic in Peru

During the first half of 2020, at least 400 innovations aimed at managing the crisis and mitigating the impact of COVID-19 were implemented in 18 Latin American countries [25]. The COVID-19 pandemic accelerated technological innovation and the adoption of policies related to telehealth, which encouraged the implementation of different telehealth programs and projects in Latin America [26]. Among the telehealth platforms developed, 1Doc3 reached 300,000 patients from all over Latin America during May 2020 and became the main telemedicine provider in Latin America [27].

Based on our literature search, we identified 12 public and private telehealth initiatives implemented in Peru. Table 2 summarizes the characteristics of the selected publications reviewed.

### 5.2. Maternal Health

A telemonitoring program was implemented at the Instituto Nacional Materno Perinatal (INMP), a national maternal-healthcare center, during the first wave of the COVID-19 pandemic in Peru (May 2020–August 2020) [28]. The telemonitoring program aimed to provide prenatal caregiving for women already registered in the institutional database or for those who requested appointments via phone calls or text messages [28]. The telemonitoring program involved appointments with an obstetrician/gynecologist via phone calls and video calls. Every consultation lasted at least 30 min, obstetric information was collected, and specific orientation about nutrition, frequent symptoms in pregnancy, and alarm signs were provided [28]. A subsequent telemonitoring or consultation with any other specialist was arranged as needed.

The INMP telemonitoring program registered 2181 telemonitoring consultations for 616 pregnant women and 544 telemonitoring consultations for puerperal women [28]. Women of low socioeconomic status represented the majority of users (49.9%). Moreover, 95% of the healthcare providers agreed that the INMP telemonitoring program was adequate when in-person visits were difficult [28].

Meza-Santibañez et al. (2021) evaluated a mixed telehealth model for prenatal care that was carried out at INMP, located in Lima, which began in August 2020 [29]. This telehealth model started with an optimization of the appointment-scheduling process (via WhatsApp). The pregnant women were asked to fill out a registration form, including the type of visit and type of insurance, before scheduling a teleconsultation [29].

The healthcare professionals who offered virtual visits (via phone calls) were selected because of the specific risk factors of contracting COVID-19; therefore, teleconsultations with the pregnant women were conducted by telephone. During the teleconsultation, complementary tests were ordered, and medications were prescribed according to the needs of the pregnant woman; above all, the healthcare professional determine whether the patient should perform the upcoming care in person or virtually [29].

The mixed telehealth model was easily adopted by the healthcare professionals as well as by the users of the system [29]. The limited access to the INMP during the first wave of the COVID-19 pandemic (due to the mandatory lockdown established by the government) was the main limitation due to the weaknesses of the public-health national referral system. The number of private and public healthcare centers was insufficient due to the high demand for face-to-face healthcare services [29].

### 5.3. Nutrition

The study carried out by Barrón and Sifuentes (2022) analyzed the main barriers to and facilitators of the implementation of a nutrition-teleguidance service in three healthcare centers located in Lima, which serve a mostly low-income population [30]. The implementation of the telehealth system was unforeseen and therefore prone to difficulties since the beginning [30]. The main barrier to the implementation of the teleguidance services that stands out was the lack of technological resources at the healthcare centers and in patients’ homes, with the lack of access to the Internet or a stable mobile line being additional noteworthy barriers. Likewise, both the health personnel and the users identified the WhatsApp social network as a facilitating factor for the nutrition-teleguidance service because it was easy to use and very accessible for users [30]. The benefits of the nutrition-teleguidance service perceived by users included flexibility in schedules, convenience in reducing travel time, a safety barrier keeping patients protected against the spread of COVID-19, and the ease of use [30].

At the Allikay Nutritional Center in Lima, a telenutrition service was implemented involving the use of information and communication technologies in the nutritional-care process [31]. Castrillón et al. conducted a retrospective study on 100 overweight or obese patients from January 2019 to March 2021. Researchers found that both the telenutrition and face-to-face services were similar in terms of weight reduction, body-mass index, waist circumference, and relative fat mass [31]. In addition, no significant differences were found between the reductions observed in all the parameters evaluated in both the telenutrition and in-person care. However, the results obtained should be considered with caution since the sample was selected by convenience; the follow-up program aftercare was carried out by email or via WhatsApp [31].

In Tarapoto, Marquez et al. [32] implemented a telehealth program for 18 weeks (August to December 2021), including university teaching staff from three regions of Peru (coast, highlands, and jungle). In the study, 78 participants were included, who evaluated lifestyle practices and beliefs and measurements of body-mass index and blood glucose before and after the program [32]. The whole training was provided through the Zoom platform by specialists in health promotion, healthy lifestyles, and disease prevention. The intervention managed to significantly reduce the body-mass index and glucose concentration as well as increase lifestyle practices and beliefs. However, it should be noted that there were many limitations in this study, such as the absence of a control group, a non-randomization process, and a non-representative sample [32].

### 5.4. Mental Health

In the field of mental health, the Hermilio Valdizán Hospital, an institution specializing in psychiatry and mental health, has been implementing telehealth services since 2018, providing coordinated teleinterconsultations with healthcare professionals from rural areas and training health personnel through teletraining and training aimed at the general population through teleinformation, tele-education, and telecommunication [33]. At the beginning of the COVID-19 pandemic in Peru, the staff used WhatsApp to coordinate appointments, a Google Form to collect general information and informed consent, and a Zoom account provided by the Ministry of Health to carry out teleconsultations, teleinterconsultations, teletraining, and teleinformation, -education, and -communication [33]. Subsequently, an Intranet—Hermilio Valdizán Hospital application and an institutional Zoom account were implemented [33].

Between March and December 2020, 57,398 virtual consultations (teleconsulting and telemonitoring) were provided, including 4411 mental-health teleorientations [33]. It should be noted that psychotherapy services aimed at children, adolescents, adults, the elderly, couples, and families, as well as psychological evaluations, were carried out totally virtually. Regarding teletraining, the Hermilio Valdizán Hospital held 29 individual presentations and 15 academic conferences; likewise, they carried out more than 30 teleinformation, -education, and -communication services that were shared through the hospital’s social networks [33].

### 5.5. Oncology

In the area of oncology, the National Institute of Neoplastic Diseases (INEN) implemented telemedicine services to provide cancer care in response to the COVID-19 pandemic. To evaluate the impact of this program, Roque et al. conducted a study about the oncological care provided during the COVID-19 pandemic [34]. Researchers reported that 16,456 teleconsultations were performed during March 2020 to February 2021. More telemedicine services were used during the first rather than the second wave. The satisfaction of the users of the telehealth services was evaluated in three aspects: the appointment-scheduling process, the telemedicine service, and the distribution of medicines and orders. For this survey, between July and October 2020, a modified version of the University of Kansas Cancer Center telephone satisfaction-survey test was applied to 5765 patients chosen randomly. A score of 4.6/5 was obtained for the appointment-request process, 4.58/5 for the telemedicine service, and 4.33/5 for the distribution of medicines; therefore, the INEN telemedicine service can be considered to have been a useful tool to maintain the continuity of cancer care for oncological patients [34].

### 5.6. Radiology

In telediagnosis, a study published by Marini et al. stated that it is possible to carry out lung-imaging diagnosis and evaluation of sequalae of COVID-19 through the use of lung-volume-sweep imaging teleultrasound performed by operators without prior training [35]. The study was carried out in five rural communities in Peru in which training was provided on how to use a teleultrasound system and perform a lung-volume-sweep imaging ultrasound. The participants had no previous experience in ultrasound. Once the images were obtained by the operators, they were sent to be evaluated by radiology specialists [35]. Of the 213 tests carried out, 202 were qualified as suitable to establish a diagnosis. Likewise, when 20% of the exams were randomly evaluated, there was a 91% concordance between the diagnoses established by the radiologists [35].

A previous telediagnosis initiative was described by Marini et al. [36], who described the experience of performing asynchronous telediagnosis for right-upper-quadrant abdominal-ultrasound examination performed by inexperienced operators. In this case, 91 of the 144 extracted images were of acceptable or excellent quality. In addition, there was 95% agreement between teleultrasound and standard of care regarding whether an exam was normal or abnormal. Finally, for the diagnosis of cholelithiasis, the sensitivity was 93% and the specificity was 97% [36].

### 5.7. Ophthalmology

In 16 Amazon villages from Alto Amazonas that did not have access to ophthalmology specialists, Nesemann et al. performed a screening for visual-health problems in adults over 50 years of age [37]. The measurement of visual acuity (VA) was performed using a VA measurement card approved by the Ministry of Health of Peru and photographs of the anterior segment of the eye using a smartphone accessory created via 3D printing. The results of the VA evaluation and photographs were evaluated by specialists remotely. As a result of the evaluation, 123 people with non-refractive visual impairment were identified, and among the most frequent diseases were pterygium, corneal opacity, and tuberculosis [37]. However, inter-rater agreement between the seven photo-graders had poor reliability for cataract (ICC 0.42) and corneal-opacity (ICC 0.37) diagnosis, moderate reliability for pterygium (ICC 0.67) diagnosis, and good reliability for phthisis-bulbi (ICC 0.76) diagnosis. Additionally, the authors pointed out that complete diagnosis of a visual impairment requires an evaluation of the posterior segment of the eye. The accessory created by 3D printing mainly proved to be easy to use by non-specialist healthcare workers. Although the cellphone accessory cannot identify the cause of visual impairment, it could be used to screen adults with visual impairment to detect potentially treatable eye conditions in areas with limited access to specialized ophthalmology services [37].

### 5.8. Other Medical Specialties

In the private sector, Huapaya-Huertas reported on the implementation process of telehealth services in eight healthcare centers in Peru, including five in Lima and one each in Arequipa, Trujillo, and Huaraz [38]. The implemented system began by obtaining an appointment that could be accessed through a mobile application, a website, and via a phone call. The patient received a notification by telephone two hours before the service. For the teleconsultation platform, an external service called DOC24 was contracted that allowed teleconsultations, which were registered through an electronic-record system [38]. Later, the patient could receive a call from the healthcare center to confirm how satisfied they were with the virtual consultation performed [38]. In the beginning, the model required health professionals to access the telehealth service only at the healthcare center; however, later, they could access the platform at home via virtual private network (VPN) access. Among the most requested specialties for a teleconsultation were internal medicine (30.6%), endocrinology (12.7%), and pulmonology (6.2%) [38].

In the public sector, Barriga-Chambi et al. evaluated the level of satisfaction of healthcare workers and patients with the telehealth service of the Hospital III Regional Honorio Delgado (Arequipa), as well as the maturity level of the telehealth service’s implementation, from October to December 2021 [39]. The authors found that non-physician professionals’ satisfaction with the telehealth service was higher than that of physicians (72.5% vs. 18.3%). In addition, 77.6% of patients stated they were satisfied with the service [39]. Regarding the maturity level (assessed using the Pan American Health Organization’s (PAHO) instrument for measuring the maturity level of healthcare institutions implementing telemedicine), the Hospital III Regional Honorio Delgado telemedicine service had 32% of its items in a null status and 40.8% in started, 25.2% in advanced, and 2% in ready conditions. In addition, although physician satisfaction was lower than that of other health professionals, patients had moderately high satisfaction [39].

## 6. Discussion

The COVID-19 pandemic brought many challenges but at the same time provided unique opportunities for the development and implementation of innovative solutions, including telehealth and telemedicine [40]. As Bouabida et al. stated, “telehealth technologies act as an effective, attractive, and affordable option, and people are often willing to use it” [41].

The telehealth policies adopted by Latin American governments to contain the spread of COVID-19 will continue to evolve in response to the needs of patients and the public and will continue to generate alternatives and innovative solutions for strengthening healthcare systems and improve the health of the population. Telehealth, at the global level, underwent exponential development during the pandemic and faced many challenges and opportunities [42], increasing the number of telehealth projects and programs from public and private entities, many of them in collaboration with different stakeholders.

### 6.1. Key Pending Challenges to Integrate Telehealth Services for Strengthening Health Systems in Peru

Like other Latin American countries, Peru faced several challenges in implementing telehealth services [6,7,43]. Among these, the digital divide (use and access) to digital media and the quality of Internet services have already been pointed out [6,7,43]. During the pandemic, it became clear that improved infrastructure in healthcare centers, including high-speed Internet connectivity, is a critical need for telehealth implementations. In addition, pending challenges include strengthening the infostructure of health-information systems, including interoperability with electronic medical records [44], and the monitoring and evaluation of the national agenda for the health sector in 2020–2025 [45] and the National Plan for Telehealth 2020–2023 in Peru [46].

During the 2020–2022 period, the number of Internet users increased in Peru, and access coverage was increased by different companies that provide those services. However, there is still a significant access gap between the country’s urban and rural population [6,7,43]. In relation to the implementation of an adequate technological infrastructure, according to the information from the National Telehealth Network, not all healthcare centers in the country were implemented under similar conditions; whereas some regions prioritized the implementation of greater resolution capacity, others focused on the implementation of the first level of care [43].

It is important to point out the role of confidentiality, privacy, and security in telehealth services. There is a critical need to establish security measures related to the storage, management, and electronic transfer of patient data [47]. In 2011, Peru issued Law No. 29733 (Personal Data Protection Law), which established that people have the right to keep their personal data protected [48]. In addition, under the above Peruvian Personal Data Protection Law, any private or public institution must obtain informed consent from the data subject to collect personal data. During the COVID-19 pandemic, Calderon et al. noted that data-protection regulations failed to provide the needed bulwarks to protect this information from improper access, undue processing, data breaches, and other types of malicious manipulation of personal data and databases [49]. Therefore, any telehealth service or telemedicine app developed in Peru must comply with the Personal Data Protection Law and other regulations established by the Peruvian Council of Ministers and the Ministry of Health.

Despite these limitations, important achievements have been reached in Latin America (including Peru), such as the development of university telemedicine networks that promote good practices for telehealth-service implementations in public and private systems. In addition, the incorporation of telehealth in the national agenda required the development and improvement of processes, regulations, and policies that facilitated the implementation and adoption of digital services. Primary-healthcare models in Peru and Latin America still face numerous challenges, including the disarticulation of the levels of care and an inefficient management of information. In this sense, it is urgent to implement a “strong, flexible, and interoperable health information system that would allow the suitable access to precise, reliable, and consistent information about the health of the population” in order to develop a digital primary-healthcare system [50].

Above all, patients’ digital-health literacy needs to be developed and reinforced, since many Peruvians adopted telehealth services for the first time. Health-related technologies such as electronic medical records and patient portals might be very useful for the prevention, diagnosis, and treatment of various diseases [5].

In the context of the pandemic, as highlighted in this article, a wide range of telehealth services was implemented in Peru in the fields of maternal health, nutrition, mental health, oncology, radiology, and ophthalmology, among others, so that healthcare institutions could provide continuity of care for patients.

Technologies such as smartphones with 3D-printed cases were developed to facilitate the remote diagnosis of eye diseases [51], and regarding digital fabrication, 3D-printed personal protective face shields and 3D-printed medical devices and accessories were donated to physicians and healthcare hospitals in several regions of Peru by private universities and other institutions [52,53].

Telediagnosis using ultrasound images proved to be effective for the diagnosis and evaluation of sequelae of COVID-19 infection [54,55], as well as for the diagnosis of eye conditions such as cataract, pterygium, corneal opacity, and phthisis [37].

The Ministry of Health has an important role to play in strengthening the National Telehealth Network according the four axes established [56] and in the promotion and financing of training programs for multidisciplinary professionals in the fields of digital health, telehealth, and data intelligence, among other topics, in collaboration with universities [57]. Expanding the healthcare workforce to accommodate digital health and developing the capacities of healthcare users in terms of health literacy, including its digital aspects, are key pending challenges in Peru and Latin America [57].

Those telehealth advances and developments evidence how the incorporation of telehealth in the national policy of a country can generate the implementation of solutions with a high impact on the health of the population and play a critical role in strengthening health-information systems.

### 6.2. Final Remarks

Although this article highlights a diversity of telehealth projects, very few have been implemented on a national scale, and very few would be sustainable on a national scale due to the lack of a digital-health ecosystem and the disproportionate access to health technologies across the population. Telehealth represents an effective and affordable option to solve population health problems, but it also implies a sociocultural change with a sociotechnical approach aimed at changing the mindset of both health professionals and users [58].

In the age of digital interdependence, it is worth pointing out that information systems for health are a “holistic process and approach for managing interoperable applications and databases that ethically process structured and unstructured data from different sectors for the benefit of public health” [59].

In addition, it is important to recognize the key role of stakeholders, health-related institutions, universities, non-governmental organizations, technical-cooperation organizations, funding agencies, and other related institutions—both public and private, national and international—in promoting the field of telehealth and digital health, also taking into account that interdependence among stakeholders is essential in the digital age given that no single institution or organization has all the required “knowledge, creativity, or human, financial, or technological resources” [60]. The role of local and regional telemedicine societies should be discussed to promote a coordinating role for developing studies, performing evaluations, and disseminating activities.

Finally, telehealth is a very promising and effective approach in the context of strengthening health-information systems in Peru (and beyond). Moreover, telehealth might contribute to improving public-health services, including in times after the COVID-19 pandemic. Despite the many challenges described in this paper, telehealth presents many opportunities and enormous potential for strengthening and improving public-health systems. Further studies are encouraged to build an evidence base of telehealth’s impact considering the clinical, organizational, socio-economic, socio-cultural, and ethical impacts and to explore innovative and cost-effective solutions for multidisciplinary public-health issues.

## Figures and Tables

**Table 1:** Access to the Internet in Peru for people 6 years of age and older based on the point of access (April–June 2022).

Point of Access to the Internet	Percentage
Only from a mobile phone	67%
At home and from a mobile phone *	18.6%
Only from home *	4.9%
At home, at work, and from a mobile phone *	3.2%
Only at Internet cafes	0.2%
Only on someone else´s home network	0.4%
Only from work	0.1%
Only at an educational facility	0.1%
Only at a different place	0.2%
In two or more locations (neither home nor mobile)	5.4%

Adapted from National Institute of Statistics and Informatics (INEI), Peru, 2022 [23]. * From home refers to a fixed internet connection at home.

**Table 2:** Characteristics of selected COVID-19-related publications reviewed.

#	Lead Author and Year	Type of Publication	Evidence Summary
Maternal Health			
1	Novoa et al., 2022 [28]	Retrospective cohort study	The authors described a telemonitoring program that was implemented at the Instituto Nacional Materno Perinatal (INMP) during the first wave of the COVID-19 pandemic in Peru. They reviewed the medical records of pregnant women who had access to the telemonitoring program in the period between May 2020 and August 2020. They also performed a satisfaction survey wherein almost all the healthcare providers agreed that the program was adequate when in-person visits were difficult.
2	Meza-Santibañez et al., 2021 [29]	Special Article	The authors described a mixed telehealth model for prenatal care that was carried out at INMP. The model optimized the appointment-scheduling process and was easily adopted by both healthcare professionals and users. The authors highlighted that the main limitation to the model of prenatal care was the accessibility of patients to the institution due to the weaknesses of the public-health national referral system.
Nutrition			
3	Barrón et al., 2022 [30]	Thesis	The authors analyzed the main barriers and facilitators for the implementation of a nutrition-teleguidance service in three healthcare centers located in Lima. The main barriers founded were lack of access to the Internet or a stable mobile line, and the WhatsApp social network was identified as a facilitating factor for the service by both health personnel and users.
4	Castrillón et al., 2022 [31]	Observational retrospective study	The authors aimed to report the effect of a telenutrition service called Allikay Nutritional Center on weight, body-mass index (BMI), waist circumference (WC), and relative fat mass (RFM) in overweight and obese adult patients. After comparing the service with in-person evaluation, they found that there were no significant differences between the changes in anthropometric parameters when comparing both systems, so they concluded that telenutrition may be regarded as an alternative to in-person evaluation.
5	Marquez et al., 2022 [32]	Pre-experimental study	The authors aimed to investigate the effect of a telehealth program intervention on lifestyle, body-mass index (BMI), and glucose concentration in university staff during the COVID-19 pandemic. Having conducted a pre-experimental study on workers of a private university in Tarapoto, the results showed an increase in lifestyle practices and beliefs, although considered unhealthy, as well as a decrease in BMI and glucose concentration. However, there were many limitations in this study, such as the absence of a control group, a non-randomization process, and a non-representative sample.
Mental Health			
6	Alva-Arroyo et al., 2021 [33]	Special Article	The authors described the telehealth activities carried out by the Hermilio Valdizán Hospital, a mental-health institution in Peru that has been implementing telehealth services since 2018. During the COVID-19 pandemic, they provided virtual consultations, including mental-health teleorientations, with psychotherapy services and psychological evaluations conducted entirely virtually.
Oncology			
7	Roque et al., 2022 [34]	Retrospective study	The authors described the impact of a telemedicine service to provide cancer care. They found that more telemedicine services were used during the first wave. In addition, they evaluated the satisfaction of users of the telehealth service on patients that were chosen randomly; as a result, they found high levels of satisfaction in the dimensions of the appointment-scheduling process, telemedicine service, and distribution of medicines.
Radiology			
8	Marini et al., 2022 [35]	Pilot study	The authors conducted a study in five rural communities in Peru to evaluate the use of teleultrasound for lung-imaging diagnosis and evaluation of COVID-19 sequalae. The participants, who had no previous experience in ultrasound, were trained on how to use the teleultrasound system and performed the lung ultrasound. The images obtained were sent to radiology specialists for evaluation. The study showed that teleultrasound performed by operators without prior training is a feasible tool for lung-imaging diagnosis and could improve access to healthcare in underserved areas.
9	Marini et al., 2021 [36]	Pilot study	The authors described an initiative for asynchronous telediagnosis of the right-upper-quadrant abdominal-ultrasound examination by inexperienced operators. Most of the images were deemed acceptable or excellent quality, and there was a 95% agreement between teleultrasound and standard of care on the normal/abnormal diagnosis. Finally, the study suggests that asynchronous telediagnosis could be a feasible option for remote healthcare access in underserved areas.
Ophthalmology			
10	Nesemann et al., 2021 [37]	Short communication	The authors measured the visual acuity and photographs of the anterior segment of the eye using a smartphone accessory created with 3D printing in 16 Amazon villages where ophthalmology specialists were not available. Specialists evaluated the results remotely and identified people with non-refractive visual impairment; however, inter-rater agreement for some diagnoses had poor to moderate reliability.
Other Medical Specialties			
11	Huapaya-Huertas et al., 2022 [38]	Retrospective study	The authors reported on the implementation of telehealth services in eight private healthcare centers in Peru, with patients being able to make appointments through a mobile application, website, or phone call. Teleconsultations were provided using an external service called DOC24 and registered through an electronic-record system. They reported that internal medicine, endocrinology, and pulmonology were among the most requested specialties for teleconsultations.
12	Barriga-Chambi, 2022 [39]	Cross sectional study	The authors evaluated the satisfaction level of healthcare workers and patients with the telehealth service of Hospital III Regional Honorio Delgado (Arequipa). They found that physician satisfaction was lower than that of other health professionals, but patients had a moderately high satisfaction level. In addition, the authors evaluated the maturity level of the telehealth-service implementation using the Pan American Health Organization’s instrument.

## Data Availability

The data presented in this study are available on request from the corresponding authors.
